# Determination of Density of Starch Hydrogel Microspheres from Sedimentation Experiments Using Non-Stokes Drag Coefficient

**DOI:** 10.3390/gels10040277

**Published:** 2024-04-19

**Authors:** Margherita Cretella, Mina Fazilati, Nedim Krcic, Ivan Argatov, Vitaly Kocherbitov

**Affiliations:** 1 Department of Biomedical Science, Malmö University, 20506 Malmö, Swedenvitaly.kocherbitov@mau.se (V.K.); 2 Biofilms Research Center for Biointerfaces, Malmö University, 20506 Malmö, Sweden; 3 Erasmus Student, University of Salerno, 84084 Fisciano, Italy; 4 Magle Chemoswed AB, 21215 Malmö, Sweden; nedim.krcic@maglechemoswed.com; 5 Institut für Mechanik, Technische Universität Berlin, 10623 Berlin, Germany

**Keywords:** starch microparticles, cross-linked starch hydrogel, sedimentation, non-Stokes drag

## Abstract

Sedimentation is an important property of colloidal systems that should be considered when designing pharmaceutical formulations. In pharmaceutical applications, sedimentation is normally described using Stokes’ law, which assumes laminar flow of fluid. In this work we studied swelling and hydration of spherical cross-linked amorphous starch microspheres in pure water, solutions of sodium chloride, and in pH-adjusted aqueous solutions. We demonstrated that Reynolds numbers obtained in these experiments correspond to the transition regime between the laminar flow and the turbulent flow and, hence, expressions based on the non-Stokes drag coefficient should be used for calculations of sedimentation velocity from known density or for assessment of density from observed sedimentation velocity. The density of starch microparticles hydrated in water was about 1050 kg/m^3^, while densities obtained from experiment with other liquids were dependent on the liquids’ densities. The data indicate that the swelling of the cross-linked starch microparticles as characterized by their densities is not sensitive to pH and salt concentration in the studied range of these parameters.

## 1. Introduction

Sedimentation is an important process that needs to be taken into consideration when designing colloidal pharmaceutical formulations [1] or hydrogel delivery systems [2,3]. The majority of such formulations are produced in forms of dispersions or emulsions where droplets or particles have a density value different from that of the liquid medium and, therefore, are prone to sedimentation. The time needed for a particle to move a distance comparable to the height of the container is an important parameter characterizing the stability of the formulation. In general, it is beneficial for formulation stability to slow down sedimentation. This can be done by increasing viscosity or matching the density of the particle and the liquid [4]. Since this is difficult to achieve in many practical situations, deviation of the particle density from the density of surrounding liquid becomes the main factor defining sedimentation behavior in the system.

Most often, the velocity of a particle due to sedimentation is described by Stokes’ law, which, however, has certain limitations, such as spherical shape of the particles and absence of particle–particle interactions. Strictly speaking, Stokes’ law should only be applied to very dilute suspensions, where particles do not affect each other, but pharmaceutical suspensions are rarely very dilute [5]. According to Stokes’ law, the drag force acting on a spherical particle is predicted to be proportional to both the particle diameter, $d_{p}$, and the particle velocity, v, with the coefficient of proportionality equal to 3πμ with μ being the viscosity of the fluid. It is also well known that its range of applicability is determined by a range of small values of the Reynolds number (the Stokesian realm is typically defined by Re<1), which is proportional to both $d_{p}$ and v as well. This explains that the particle size is the primary controlling parameter when the properties of the fluid are fixed.

Another potential limitation deals with typical sedimentation velocities. Stokes’ law is valid at relatively low velocities of the spheres, while at higher velocities, a more complex approach based on Reynolds numbers should be used [6]. A large difference in the densities of the particle and the liquid, low viscosity, and large particle sizes promote high sedimentation velocities. Fortunately, particle sizes in pharmaceutical dispersions are very small (typically in the range of micrometers), which results in low sedimentation velocities and hence applicability of Stokes’ law. On the other hand, there are several existing and emerging types of pharmaceutical formulations, medical devices, and biotechnological processes where particle sizes can be substantially higher. One example is amorphous cross-linked starch microspheres [7,8]. They can be used as a medical device for acceleration of wound healing by promoting hemostasis and in drug delivery applications. Biodegradability [9,10] is especially beneficial for drug delivery systems since degradation to simpler non-toxic substances reduces the environmental impact and risk of side effects.

The size of these particles is typically in the range of hundreds of microns and in some cases over 1 mm. Another example is alginate beads [11,12] that can be used in controlled delivery applications. Large particles are relevant for three-dimensional (3D) cell culture [9,10] and food technology [13].

In the cases where the particle size is relatively large, the applicability of a Stokes’ law-based approach for description of sedimentation should be checked. This is important not only for estimation of stability of certain dispersion, but also due to the fact that sedimentation rate can be used for assessment of the particle size when the density is known or, alternatively, calculation of density if the particle size can be measured by an independent method.

In this work, we test the applicability of Stokes’ law for description of the sedimentation rate of cross-linked starch microparticles [7,8] and propose a method for practically accurate calculation of their densities. Due to their nearly perfectly spherical shape in the hydrated state and a large size that can be directly measured using optical microscopy, these particles are a perfect model objects for such study. The results can be generalized to other systems relevant for pharmaceutical and food industries.

## 2. Materials and Methods

### 2.1. Materials

Degradable starch microspheres (DSMs) were produced by Magle Chemoswed AB (Malmö, Sweden), using a water-in-oil emulsion cross-linking (polymerization) process involving hydrolyzed potato starch and a cross-linking agent. In this project, the DSM particles with an average particle diameter of 580 µm belonging to the DSM-D batch [8] were used. Among amorphous degradable starch microspheres, the DSM-D particles have the largest size, which facilitates optical tracing of their positions.

Milli-Q water was used as a reference fluid in the hydration and sedimentation process of degradable starch microspheres (DSM). The Milli-Q water used in this work was produced using PURELAB flex (ELGA, High Wycombe, UK). While pure water was used as a test fluid to study applicability of the sedimentation laws, we in addition tested effects of salt and pH on swelling and sedimentation of DSM.

Two different types of salt solutions were also used as reference fluids in the swelling and sedimentation experiments with degradable starch microspheres (DSMs). The salt solutions were prepared by dissolving NaCl in Milli-Q water. The concentrations of NaCl of 1 *w*/*v*% and 6 *w*/*v*% (weight per volume of solvent percentage concentration) were used, which corresponds to 0.99 wt% and 5.66 wt% respectively.

Swelling and sedimentation of DSM were also tested in acidic and basic solutions. The acidic solution was prepared by adding drops of 0.1 M HCl solution to Milli-Q water; drops were added so that the acidic aqueous solution reached the pH of 4. The basic solution was prepared by adding drops of 0.1 M NaOH solution to Milli-Q water. The drops were added until the pH of the aqueous solution reached the value of 10.

### 2.2. Optical Microscopy

An optical microscope OPTIKA B-383PL equipped with a digital camera (Optika Italy, Ponteranica, Bergamo, Italy) was used to determine the size of the starch microspheres. Through this, it was possible to investigate the morphological changes and swelling of the starch microparticles once they were added in an aqueous liquid.

In the beginning, dry starch microspheres were placed on a microscope slide and photographed to evaluate their dry diameter, then the microspheres were added inside a petri dish containing the aqueous liquid. At the end of the hydration process, microscopy images of the hydrated starch microspheres surrounded by an aqueous liquid in a Petri dish were captured in order to measure the diameter in the hydrated state.

### 2.3. Swelling of Starch Microparticles

Degradable starch microspheres (DSMs) were added inside a Petri dish containing an aqueous reference liquid. Care was taken to ensure that the starch microspheres were completely immersed in the liquid in order to allow uniform hydration at every point of the particle. Water absorption results in rapid and substantial swelling of the starch microspheres. To be sure that the starch microparticles had achieved complete hydration, they were left in the liquid for 2 h. At the end of the swelling process, it was observed under an optical microscope whether the microspheres in the presence of water changed their shape, size, and overall appearance.

Since during hydration and swelling, DSM particles might release small amounts of low-molecular-weight substances [8], the pH values of both the acidic and basic solutions used in the DSM hydration and the acidic and basic solutions used in the sedimentation experiments were checked using a pH meter. In both cases, no change in the pH of the acidic and basic solutions that came in contact with the DSMs was detected.

The volumetric swelling ratio $r_{sw}^{vol}$ of each starch microsphere was determined by dividing the cubed radius of the fully swollen particle by the cubed radius of the same particle before swelling (in the dry state):

(1) $$r_{sw}^{vol}=\frac{r_{h}^{3}}{r_{d}^{3}}$$

where $r_{h}$ is the radius of the hydrated particle, and $r_{d}$ is the radius of the same particle in the dry state.

### 2.4. Sedimentation Experiments

Sedimentation is the process of downwards movement of particles of solid or liquid material dispersed in a fluid. In this work, the microspheres used in the sedimentation process were first fully hydrated and swollen in the same fluid.

The experiment was performed using a 50 mL cylinder filled to the brim with an aqueous reference fluid. To start a sedimentation experiment, a single particle was put on the surface of the aqueous liquid inside the cylinder. The sedimentation process was filmed by a cell phone (iPhone 12). Both the 50 mL cylinder and the cell phone were placed on a 3 cm high platform and separated by a distance of 25 cm.

The sedimentation rate v was calculated from the travelled distance ∆x and the time ∆t measured for each single particle:

(2) $$v=\frac{∆x}{∆t}$$

To obtain an accurate measure of time it was necessary to divide the movie obtained from the cell phone into frames; as the video recorded was high definition at 1080p HD/30 fps, this means that every 30 frames corresponded to 1 s. Based on the number of frames obtained it was therefore possible to have an accurate measure of the time it takes for the particle to travel a certain distance. A discussion of the method’s accuracy is provided in [14] and Appendix A.

## 3. Results and Discussion

### 3.1. Particles Hydration and Swelling in Milli-Q Water

**Figure 1:**
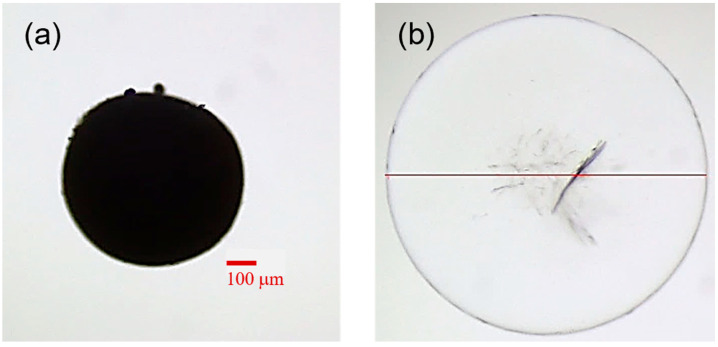
Optical microscopy images of a dry starch microsphere (**a**) and the same particle after hydration in MQ water (**b**). The line in figure (**b**) illustrates calculation of the particle diameter.

Degradable starch microspheres (DSMs) analyzed under an optical microscope in the presence of milli-Q water reveal different shapes, sizes, and overall appearances compared to the same microspheres in the absence of water. What is immediately visible under the microscope is that dry starch microspheres are dark (Figure 1a) while hydrated ones are transparent (Figure 1b). In this work, microparticles belonging to DSM-D batch were analyzed. Under the optical microscope it was possible to detect the process of hydration of each particle, and once placed in milli-Q water they become completely transparent. This indicates that the particle is uniformly hydrated. More details about the changes occurring in the DSM particles upon hydration can be found in a recent publication from our lab [8].

Another observation important for sedimentation deals with the shapes of the particles. While in the dry state many of the studied particles have imperfections in the general shape and sometimes a certain degree of surface roughness, in the hydrated state they have almost perfect spherical shape. The spherical shape of the particles is an important prerequisite for application of Stokes’ equation and for understanding of possible reasons for deviations from it.

**Figure 2:**
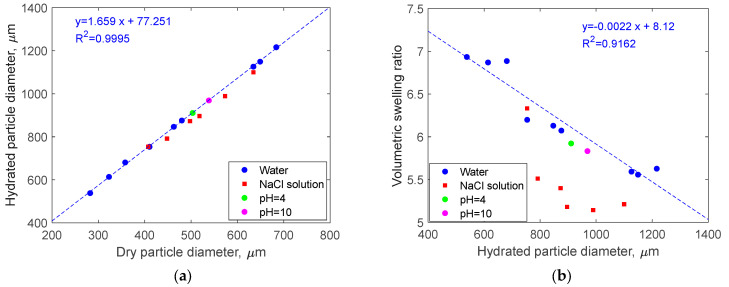
Comparison of the DSM diameter (µm) before and after contact with liquids specified in the legend (**a**) and the volumetric swelling ratio as a function of hydrated particle diameter (**b**). The linear fits are made using the data for MQ water only.

Moreover, DSMs in the presence of water undergo a swelling process that significantly changes the size of the microspheres. The size of hydrated particles as a function of the same particle size in the dry state is shown in Figure 2 and Table 1. Clearly, an excellent linear correlation is observed (Figure 2a). The trend line, however, does not start from the origin point (0, 0), but rather closes the y-axis at a positive value.

By calculating the volumetric swelling ratio of each particle, it was possible to note that the volumetric swelling ratio was somewhat lower for larger particles. The reason for this is not completely clear, but it might either be a result of a difference in distributions of cross-links and pores in the starch microparticles or caused by uncertainties of diameter measurements (especially in the dry state, see Figure 1a), or caused by variation in porosity in the dry state and presence of trapped solvent [8].

### 3.2. Sedimentation Experiments in Milli-Q Water

**Figure 3:**
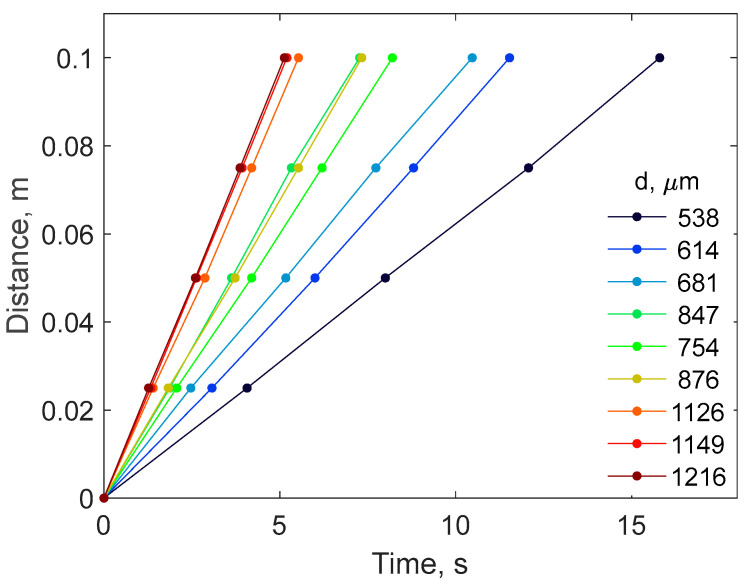
DSM particle coordinates as a function of time for the sedimentation in milli-Q water. The legend shows particle diameter.

From the sedimentation experiment, it was possible to measure the steady-state sedimentation velocity of the particles. In particular, the velocities of nine particles were analyzed, taking into account the distance the particles traveled inside the cylinder and the time it took them to reach the bottom. The speed of the particles was calculated considering a base distance of 0.10 m travelled by the particles inside the lower part of the cylinder, where the particle practically approaches the steady state. Figure 3 shows that the velocity at which individual DSM particles settle in the aqueous liquid inside the cylinder is constant, which confirms that the steady-state velocities are achieved. On the other hand, the observed velocities depend on the particle size. In particular, the larger the particle, the steeper the slope of the straight line. The obtained values of velocities are shown in the Supporting Information (Table S1).

### 3.3. Particle Density—Milli-Q Water

**Figure 4:**
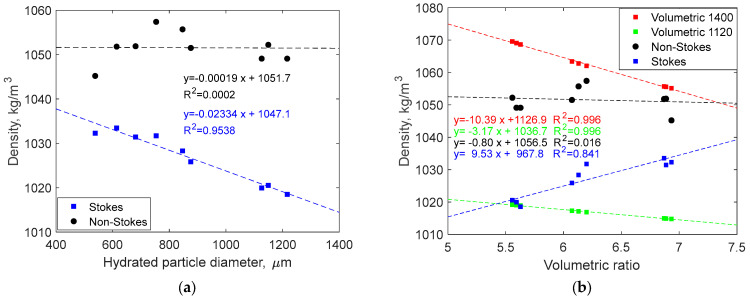
DSM particle density as a function of hydrated particle diameter (µm) in milli-Q water (**a**) and as a function of volumetric swelling ratio (**b**). The numbers in the legend specify DSM density used in calculations.

The sedimentation velocities of the particles obtained as described above can be used to calculate the density of the microspheres. The microparticles during the sedimentation experiment are subjected to different forces that push them upwards, like the resistance force and buoyancy force, and downwards, like the gravity force. Utilizing the action of these forces, it is possible to assess the density of the particles. In the case of laminar flow, the density can be calculated using Stokes’ law:

(3) $$\rho_{p}=\frac{9\mu v}{2gr_{p}^{2}}+\rho_{f}$$

where v is the settling velocity of the particle (m/s), g is the acceleration of gravity (m/s^2^), ${r_{p}=d}_{p}/2$ is the radius of the particle (m), and $\rho_{f}$ and $\rho_{p}$ are the densities of the fluid and the particle, respectively (kg/m^3^). The results of calculation of density using this approach are shown in Figure 4 (blue symbols). Clearly, the obtained density values are dependent on the particle size, which can be illustrated by high R^2^ value. This dependence should not be expected since the particles in the liquid state consist of the same material (the differences arising from dry porosity and trapped air are relevant for the solid state). This indicates the inapplicability of Stokes’ law for describing the sedimentation of starch particles of this size and density.

In order to have an accurate measure of particle density, it is of pivotal importance to predict the flow characteristics in different situations that the relative fluid flow can assume; for this purpose, it is necessary to calculate the Reynolds number [15]:

(4) $$Re=\frac{\rho_{f}\;d_{p}v}{\mu }$$

where $\rho_{f}$ is the density of the fluid (kg/m^3^), $d_{p}$ is the diameter of the particle (m), *v* is the particle velocity (m/s), and *μ* is the viscosity of the fluid (Pa∙s). At low Reynolds number values, i.e., Re < 0.5 (exact threshold values can vary from publication to publication), the fluid exhibits a laminar flow, and in this case the density of the microspheres can be calculated using Stokes’ law (Equation (3)).

At higher values of Reynolds number (Re > 0.5), the fluid flow is non-laminar. In particular, Reynolds numbers higher than 750 correspond to a Newtonian or turbulent regime [16], and the values between 0.5 and 750 correspond to a transition regime. In the case of relative flow in a transition regime, it is possible to calculate the density of microspheres using another approach [17], taking into account the drag coefficient $C_{D}\left(Re\right)$ as

(5) $${\rho_{p}=\rho }_{f}\left(1+\frac{3v^{2}}{4d_{p}g}C_{D}\left(Re\right)\right)$$

The above equation was derived by evaluating the drag force in the form $F_{drag}=\left(\frac{1}{2}\right)\rho_{f}v^{2}A_{p}C_{D}\left(Re\right)$, where $A_{p}=\pi r_{p}^{2}=\frac{\pi d_{p}^{2}}{4}$ is the projected area of the spherical particle. In the steady-state settling of a particle, the drag force balances the particle weight $m_{p}g$ minus the buoyancy $V_{p}\rho_{f}g$, where $m_{p}=\rho_{p}V_{p}$ is the mass of the particle, and $V_{p}=\left(\frac{4\pi }{3}\right)r_{p}^{3}=\left(\frac{\pi }{6}\right)d_{p}^{3}$ is the particle volume.

The following simple dependence (Schiller—Naumann model) of the drag coefficient $C_{D}\left(Re\right)$ on the Reynolds number is usually recommended for the transition range [6]

(6) $$C_{D}\left(Re\right)=\frac{24}{Re}\left(1+0.15\cdot {Re}^{0.687}\right)$$

The DSM-D particles in the sedimentation experiments showed values of the Reynolds number corresponding to the transition range in all cases (Table 2); thus, the density of the particles can be and was calculated by means of Equation (5).

The obtained density values had a mean value of 1051.5 kg/m^3^, with the standard deviation of 3.6 kg/m^3^ (or rounding to one significant digit, 1052 ± 4 kg/m^3^). The dependence of particle density on the hydrated particle diameter is shown in Figure 4 (black circles). The first point on the plot substantially deviates from the rest; on the other hand, its deviation from the density mean value is only 0.6%. Unlike the case of a Stokes’ law-based approach, the dependence on the particle diameter is flat and a very low value of R^2^ further confirms the absence of correlation with the particle size. The two linear regressions presented in Figure 4 suggest that at lower practice sizes (tens rather than hundreds of microns), both methods would provide relatively similar results. Overall, the results presented here demonstrate a good consistency and a practical accuracy of the method based on the non-Stokes drag coefficient.

Alternatively, the density of starch microparticles can be calculated from the volumetric swelling ratio $r_{sw}^{vol}$ if the dry particle density $\rho_{dp}$ is known:

(7) $$\rho =\rho_{f}+\frac{\rho_{dp}-\rho_{f}}{r_{sw}^{vol}}$$

Recently, the density of DSM particles was measured by He-pycnometry [8] but the results strongly vary from batch to batch (from 1120 to 1400 kg/m^3^) because of particles’ internal porosity in the dry state. The presence of dry porosity is clearly visible in SEM images [8]. Since the lower value is more strongly affected by the dry porosity in starch microspheres (that disappears during hydration), we suggest that the value of 1400 kg/m^3^ is more appropriate for these calculations. However, results based on both density values are presented in Figure 4b for comparison. Note that Equation (7) is non-linear with respect to swelling ratio, hence the dry density values cannot be recovered from the linear regressions presented for volumetric data (red and green symbols in the figure). The dry density variation introduces a large uncertainty in calculations using Equation (7). In contrast, the method based on sedimentation does not have this disadvantage because it does not rely on the particle properties in the dry state. Notably, only the density values calculated using a non-Stokes approach exhibit absence of correlation with the formal values of volumetric swelling ratio. The results based on volumetric and Stokes approaches show opposite trends (descending and ascending respectively). While the trends in these two cases are caused by different reasons, absence of consistent results further illustrates the idea that the two methods are less appropriate for accurate calculations of density.

### 3.4. Hydration and Swelling of Starch Particles in Salt Solutions

In this part of the work, we considered another aqueous reference liquid: a saline solution. The starch microspheres belonging to the DSM-D batch have been hydrated by immersing some of them in a 1% NaCl salt solution and others in a 6% NaCl salt solution.

In both cases—after the hydration process of 2 h—under the optical microscope, it was possible to observe that the hydrated starch microspheres were completely transparent, as well as when we immersed the particles in milli-Q water (see Section 3.1). The analyzed particles, also in this case, had swollen such that the diameter of the hydrated starch microspheres was almost twice as large as those in the dry state: the obtained values of the diameters are shown in Table S2.

The typical volumetric swelling ratios in salt solutions were somewhat lower than in MQ water, see Figure 2b, red symbols. By calculating the volumetric swelling ratio of each particle, it was possible to note that the volumetric swelling ratio is lower for larger particles. This correlation was seen in both particles immersed in 1% NaCl salt solution and those immersed in 6% NaCl salt solution (Table S2).

Through sedimentation experiments, we measured sedimentation velocities of the particles in the salt solutions. After hydrating the starch microparticles, the particles were individually subjected to sedimentation experiments. The particles hydrated with a 1% or 6% NaCl solution were added into the cylinder containing the same salt solution to perform the sedimentation experiment. The observed stationary state velocities at which individual particles settle in both salt solutions were dependent on particle size (Table S3).

**Figure 5:**
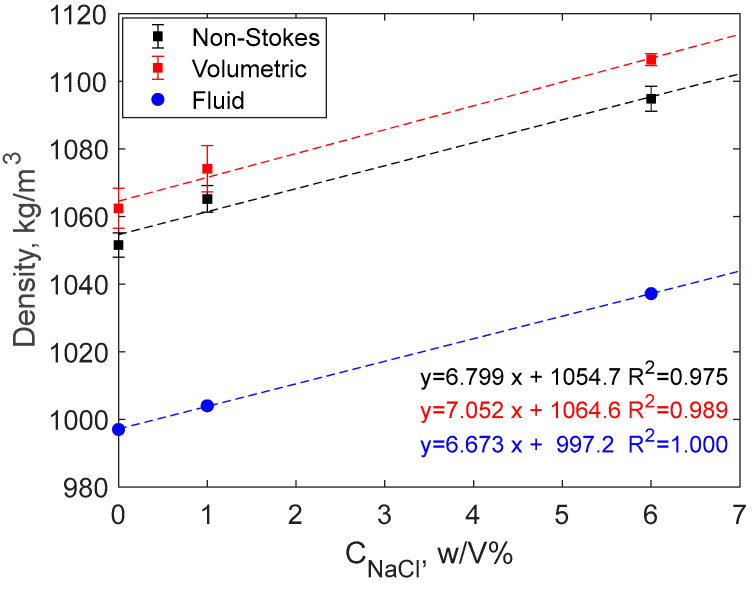
DSM average particle density as a function of salt concentration. The error bars are standard deviations.

The analysis shows high Reynolds number values (Re > 10); therefore, the density of the starch microspheres was calculated using Equations (4)–(6) using literature values for the viscosity (0.00089 and 0.000972 Pa∙s) and density (1004.03 kg/m^3^ and 1037.19 kg/m^3^) of 1 and 6 *w*/*v*% salt solutions, respectively [18,19]. The results show an increase in particle densities compared to the values obtained using milli-Q water. The main part of this increase comes, however, from the increase of the density of fluid. This is clear from Figure 5, where the three linear regressions have very similar slopes.

A smaller part of the density increase arises from reduced swelling of the starch microparticles in the presence of salt. This can be seen from three related observations. Firstly, the swelling ratio is lower in the presence of salt, as seen from Figure 2b. Secondly, the slope of the linear regression describing the fluid density is lowest among the three data sets in Figure 5. Finally, Table 3 shows that the average increase in density compared to the fluid is highest for the salt solutions. Nonetheless, the density increase compared to particles in pure water is less than 10 kg/m^3^.

### 3.5. Starch Particles Hydration, Sedimentation Experiments, and Particle Densities at Controlled pH

In order to evaluate the effect that DSMs could have on the pH of reference fluids, analyses were carried out taking into account both an aqueous acidic solution of HCl at pH 4 and an aqueous basic solution of NaOH at pH 10. In both solutions, the DSMs subjected to the 2 h hydration process once again under the optical microscope were showed to be completely transparent starch microspheres—similar to the cases when DSM particles were immersed in milli-Q water or in salt solutions (Section 3.1 and Section 3.4). The obtained swelling ratios are shown in Table 4, and the volumetric density values (based on the starch density of 1400 kg/m^3^) are 1065.1 and 1066.1 kg/m^3^ for the acidic and basic solutions, respectively.

After hydrating the starch microparticles in an aqueous acidic HCl solution at pH 4 or in a basic aqueous NaOH solution at pH 10, the same particles were tested in the sedimentation experiments by placing them in a cylinder containing the same fluid.

The hydrated particles subjected to the sedimentation process in the acidic solution showed a high Reynolds number value (see Table 5), so the density of the starch microspheres was calculated using Equations (4)–(6). The results show a particle density value of 1053.4 kg/m^3^, close to that obtained using only milli-Q water. Also, the particles hydrated and subjected to the sedimentation process in the basic solution show a high Reynolds number value, so the density of the starch microspheres was calculated using Equations (4)–(6). The results show a particle density value of 1049.9 kg/m^3^, again close to that obtained using milli-Q water.

Unlike experiments with MQ water and NaCl solutions, only single measurements were taken for both acidic and basic conditions, which does not allow for a statistical comparison of the results. Hence, more experiments are needed to draw quantitative conclusions on the effect of pH on starch microparticle swelling, especially across a broader pH range.

### 3.6. The Effect of Nonlaminar Flow in Sedimentation of Starch Particles

The result presented above clearly show that non-laminar-flow effects are very important for the description of starch microparticles sedimentation processes. This can further be illustrated by calculations of sedimentation velocities predicted by Stokes’ law and non-laminar-flow equations (Equations (5)–(6)). Figure 5 shows the calculated velocities of spherical particles of different diameters relevant for colloidal systems in water. In the case of Equations (4)–(6), the velocities were calculated numerically in MATLAB using liquid density of 997 kg/m^3^ and the particle density of 1050 kg/m^3^. The results demonstrate that for small particles with diameters less than 0.3 mm, the velocities calculated by the two methods are similar. In contrast, for large particles the discrepancy is substantial and for 2 mm particles it reaches the factor of 3.6. At these conditions, the Reynolds number can reach a value close to 64 (Figure 5). It should be noted that these calculations use the density of 1050 kg/m^3^ typical for strongly hydrated particles to a large extent consisting of water. For dry or crystalline/semi-crystalline starch-based materials, which have much higher density, the effect of non-laminar flow is expected to be even stronger.

### 3.7. Effects of Salt and pH on Swelling of Cross-Linked Starch Microparticles

In pharmaceutical applications, pure water is seldom relevant as a medium for drug delivery vehicles. Both drug absorption and release are performed at certain pH values and salt concentrations appropriate for a particular combination of the drug and the carrier particle. In many cases, these conditions can affect the properties of the drug delivery vehicles, including their swelling ratios and densities.

**Figure 6:**
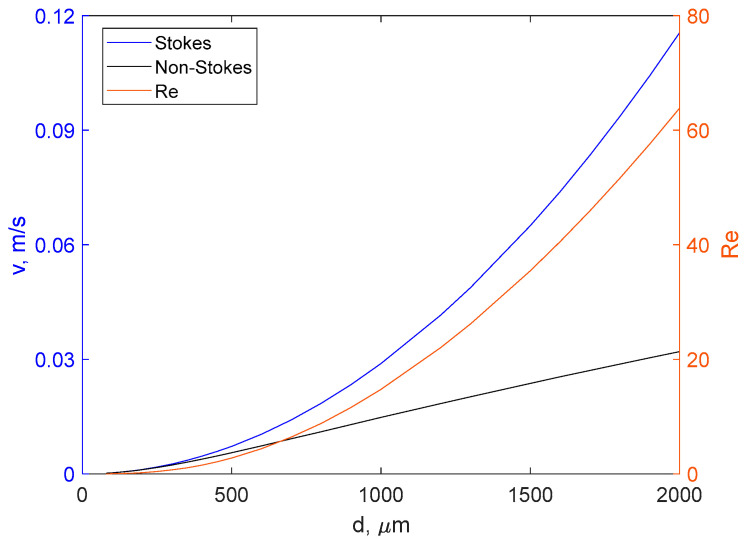
The sedimentation rate of spherical particles in water calculated from Equations (4)–(6) and Stokes’ law using the density value of 1050 kg/m^3^ (left axis). Reynolds number as a function of particle size calculated for the same density (right axis).

In this work, the sedimentation of DSM particles was analyzed in several reference fluids: milli-Q water, NaCl salt solution, acidic HCl solution, and basic NaOH solution. Taking together the density data obtained as a function of NaCl concentration in milli-Q water, we find a linearity between obtained particle density values and the NaCl concentration in milli-Q water (Figure 6). In particular, the higher the salt concentration in the reference fluid, the higher the density of the tested DSM particles.

Taking into account the densities of the reference fluids—used both in the DSM hydration process and in the sedimentation experiments—it was possible to calculate the difference in densities of the particle and the fluid ∆*ρ* (Table 3). This analysis shows that the ∆*ρ* values are relatively similar for all studied cases—pure water, basic and acidic solutions, and two salt solutions. This demonstrates that DSM particles are swollen/hydrated to a relatively similar extent in all these cases. Keeping in mind that colloidal particles are seldom electrically neutral, one could expect a screening effect upon addition of salt, which would increase the density of hydrated particles. Moreover, increase in pH may promote negative charge in the material and, hence, increase swelling and decrease the density. From Table 3, it follows that, indeed, the ∆*ρ* values observed in salt solutions are highest while the ∆*ρ* value in basic solution is lowest. These trends are, however, very small and comparable with standard deviations of the measurements. It is instructive to compare this to polyelectrolyte-based charged hydrogels [20,21], where degree of swelling can change by an order of magnitude due to pH and salt effects. This comparison shows that the DSM particles demonstrate robust swelling properties that are not sensitive to the parameters of the aqueous fluid such as pH and ionic strength.

## 4. Conclusions

We performed single-particle hydration and sedimentation experiments with cross-linked starch microspheres. It has been shown that sedimentation analysis of starch-related materials should be based on a non-Stokesian approach. The results demonstrate that effects of non-laminar flow should be taken into account when considering sedimentation of starch microspheres with diameters higher than 0.3 mm. The observed Reynolds numbers have values much higher than 0.5 and, hence, non-Stokes drag coefficients should be used for calculations of sedimentation velocities when densities are known or densities are evaluated from velocities values. As a result, we developed a simple method for experimental density determination of fully hydrated starch microparticles.

The densities of hydrated starch microspheres calculated from the sedimentation experiments are found to be close to 1050 kg/m^3^ for hydration in pure liquid water. When the particles are swollen in other aqueous fluids, their densities depend on the density of the fluid. Based on the calculated densities, we conclude that the cross-linked amorphous starch microparticles demonstrate swelling properties (with the volume swelling ratio ranging from 5 to 7) that are not sensitive to pH and salt content of the aqueous liquid in the studied ranges of these parameters—a property that can be useful for drug delivery applications.

## Figures and Tables

**Table 1:** Overview of DSM changes before and after milli-Q water contact.

Particle	Dry Particle Diameter (µm)	Hydrated Particle Diameter (µm)	Volumetric Swelling Ratio (µm^3^/µm^3^)
P1	282.3	538.3	6.94
P2	323.0	614.0	6.87
P3	357.9	680.9	6.88
P4	462.7	846.8	6.13
P5	410.3	753.7	6.20
P6	480.1	875.9	6.07
P7	634.4	1126.1	5.60
P8	648.9	1149.4	5.56
P9	683.8	1216.3	5.63

**Table 2:** DSM particle densities, Reynolds numbers, and drag coefficients in milli-Q water.

	Particle Density (kg/m^3^)	Reynolds Number, Re	Drag Coefficient, *C*_D_
P1	1045.2	3.76	8.76
P2	1051.8	5.79	6.22
P3	1051.9	7.45	5.14
P4	1055.7	13.04	3.45
P5	1057.4	10.19	4.09
P6	1051.5	13.30	3.4
P7	1049.1	22.46	2.43
P8	1052.2	22.47	2.3
P9	1049.1	26.52	2.19

**Table 3:** DSM average particle density (kg/m^3^) relative to bulk densities (kg/m^3^).

	Average Particle Density (kg/m^3^)	Fluid Density (kg/m^3^)	Density Difference ∆*ρ* (kg/m^3^)
Milli-Q water	1051.5	997.0	54.5
Acid Solution HCl pH = 4	1053.4	997.0	56.4
Basic Solution NaOH pH = 10	1049.9	997.0	52.9
NaCl 1%	1065.2	1004.0	61.2
NaCl 6%	1094.8	1037.2	57.6

**Table 4:** Overview of DSM changes before and after contact with acidic and basic solutions.

		Dry Particle Diameter (µm)	Hydrated Particle Diameter (µm)	Volumetric Swelling Ratio (µm^3^/µm^3^)
Acid Solution HCl pH = 4	PAS ^a^	503.4	910.8	5.92
Basic Solution NaOH pH = 10	PBS	538.3	969.0	5.83

^a^ PAS stands for particle in acid solution, PBS—particle in basic solution.

**Table 5:** DSM particle densities, Reynolds numbers, and drag coefficients in acid and basic solutions.

		Particle Density (kg/m^3^)	Reynolds Number, Re	Drag Coefficient, *C*_D_
Acid Solution HCl, pH = 4	PAS	1053.4	14.9	3.15
Basic Solution NaOH, pH = 10	PBS	1049.9	16.3	2.97

**Figure A1:**
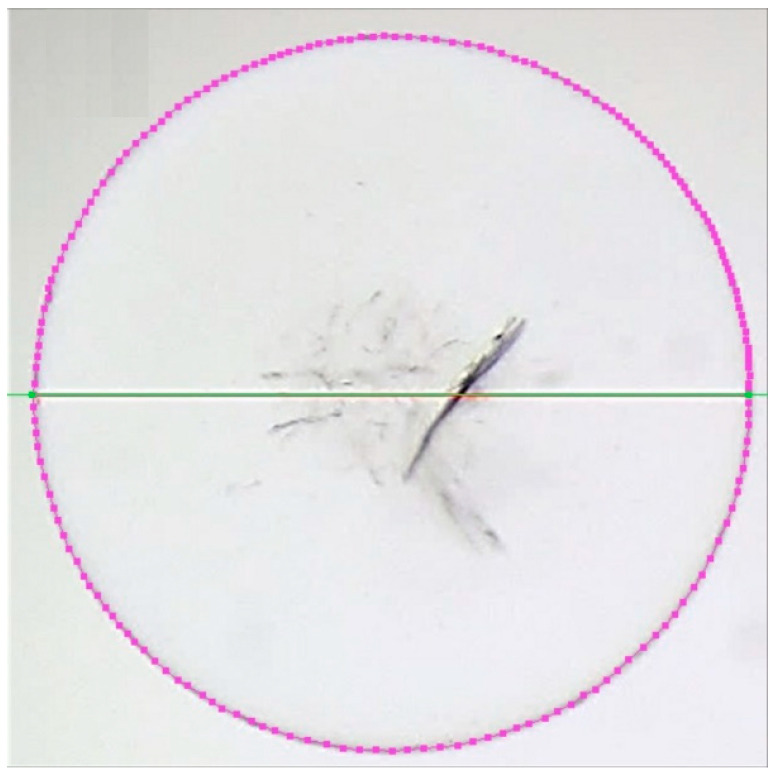
Digitized image of a hydrated starch microsphere (the same as in Figure 1b).

**Figure A2:**
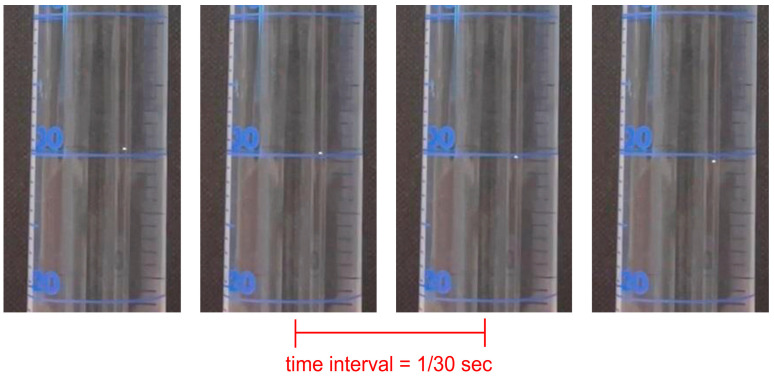
Successive frames of the experiment video.

## Data Availability

The raw data supporting the conclusions of this article will be made available by the authors on request.

## Supplementary Materials

The supporting information can be downloaded at: https://www.mdpi.com/article/10.3390/gels10040277/s1. Figure S1: Comparison of DSM volumetric swelling ratio (µm^3^/µm^3^) with the diameter (µm) of the hydrated starch microsphere in a NaCl 1% salt solution and in a NaCl 6% salt solution; Figure S2. Travelled distances in a NaCl 1% salt solution (a) and in a NaCl 6% salt solution (b); Table S1. DSM average sedimentation velocity in MQ water; Table S2. Swelling of DSM particles in salt solutions; Table S3. Sedimentation of DSM particles in salt solutions. Average velocities, Reynolds numbers and drag coefficients; Table S4. DSM average velocity in acid and basic solutions.

## Appendix A. An Estimate of the Method’s Accuracy

There are three main sources of errors of the applied method, which is based on Formula (5), namely: (1) Evaluation of the particle diameter $d_{p}$; (2) Evaluation of the particle velocity v; (3) Accuracy of the Schiller–Naumann (SN) model for the drag coefficient $C_{D}\left(Re\right)$.

First of all, it should be emphasized that the SN model is empirical in the sense that the coefficient 0.15 and the exponent 0.687 in the braces on the right-hand side of Equation (6) were determined by fitting experimental data. The accuracy of the SN model is estimated against the Clift–Grace–Weber (CGW) correlations of drag coefficient $C_{D}$ with Reynolds number Re [22], which is widely accepted [23] as one of the best approximations for the standard drag curve $C_{D}\left(Re\right)$. We note that the Schiller—Naumann model fits the data in the intermediate range (0.3 < Re < 500) with an accuracy of around ±7% [24]. In our tests, the particle Reynolds number falls into the range 3 < Re < 30, where the absolute percentage error of the model is less than 3%.

Second, the accuracy of determining the particle diameter is quite high. The visual diameter estimate was verified by digitizing the particle contour (see Figure A1) and fitting a circle to the set of points. The difference between the results of the two methods was found to be less than 0.1%. It is also of interest to note that the relative percentage deviation of the particle shape from ideal sphericity was found to be less than 0.6%. Thus, it will be safe to assume that the error of the particle diameter estimate is less than 1%.

Third, the most intriguing aspect of the error estimate concerns the evaluation of the particle velocity, which was performed by using a mobile phone camera. The sedimentation rate v is calculated by means of Formula (2), which represents a ratio of the traveled distance ∆x to the corresponding time ∆t. The mobile phone-based method employs the discrete time moments separated by the basic (smallest) time interval, ∆τ, equal to 1/30 s. Therefore, practically speaking (that is, assuming that the time moments are measured practically exactly—the error of time measuring is negligible compared to other involved errors), the particle velocity error will be determined by the accuracy of evaluating the travelled distance. The latter quantity was also recorded in a discrete way associated with the thick volume marks printed on the glass cylinder, which are separated by a distance of 2.5 cm.

Figure A2 shows four successive frames from one experiment video illustrating how a moving particle crosses one of the middle-volume marks. For the basic time interval $∆\tau =\frac{1}{13}$ s, the particle moving with a velocity v will travel the distance v∆τ. This means that the more slowly the particle moves, the more accurately can be determined its position with respect to the mark. Let us estimate the maximum possible error by taking the following upper bound for the observed sedimentation rates: $v_{*}=0.02$ m/s. The corresponding distance increment (i.e., the travelled distance between the particle position from the two successive frames) under uniform movement will be $v_{*}∆\tau =\frac{2}{3}$ mm, which is less than 0.67 mm, but it is comparable to the half-thickness of the mark. Therefore, in this extreme case, the absolute error of determining the position, ∆l, will be less than $\frac{v_{*}∆\tau }{2}$, because we have to choose one of the two frames closest to the mark position. Thus, the discrete travelled distance can be estimated with the maximum error of 2∆l ≤ 0.67 mm. For the distances of 2.5 cm, 5 cm, 7.5 cm, and 10 cm this brings the maximum relative absolute percentage errors to 2.68%, 1.34%, 0.89%, and 0.67%, respectively.

Thus, altogether, the developed method produces a maximum relative absolute percentage error not larger than about 2–3%, mainly due to the inherent uncertainty of the drag coefficient model. One should note that this error is related to the deviation of the particle density from the fluid density, see Equation (5) in the article main text. The error in the particles’ density values calculated using this method is an order of magnitude lower. For example, when the density is close to 1050 kg/m^3^, the typical error is close to 1 kg/m^3^ or 0.1%.

It is pertinent to note here that smartphones are becoming an integral part of modern laboratory research due to rapid development of their technical parameters, versatility, portability, and advanced features [25].

Finally, we note that the effect of the specific media conductivity on the electrical force, which is proportional to the particle charge [26], is not taken into account because the starch particles are neutral.
