# Supplementary material for: Determination of Density of Starch Hydrogel Microspheres from Sedimentation Experiments Using Non-Stokes Drag Coefficient
Source: Gels. 2024 Apr 19;10(4):277. doi: 10.3390/gels10040277 (PMC11049465; doi:10.3390/gels10040277)
Supplement: Supplementary file 1 [file gels-10-00277-s001.zip › gels-2960898-supplementary.pdf]

Supporting info

Determination of Density of Starch Hydrogel Microspheres from Sedimentation Experiments Using Non-Stokes Drag Coefficient

Margherita Cretella [1,2,3], Mina Fazilati [1,2], Nedim Krcic [4], Ivan Argatov [5,*] and Vitaly Kocherbitov [1,2]

[1] Department of Biomedical science, Malmö University, 20506 Malmö, Sweden; vitaly.kocherbitov@mau.se (V.K.)
[2] Biofilms Research Center for Biointerfaces, Malmö University, 20506 Malmö, Sweden
[3] Erasmus Student, University of Salerno, 84084 Fisciano, Italy
[4] Magle Chemoswed AB, 21215 Malmö, Sweden; nedim.krcic@maglechemoswed.com
[5] Institut für Mechanik, Technische Universität Berlin, 10623 Berlin, Germany
* Correspondence: ivan.argatov@campus.tu-berlin.de

S1. Additional graphs and tables

S1.1. Volumetric swelling ratios in salt solutions

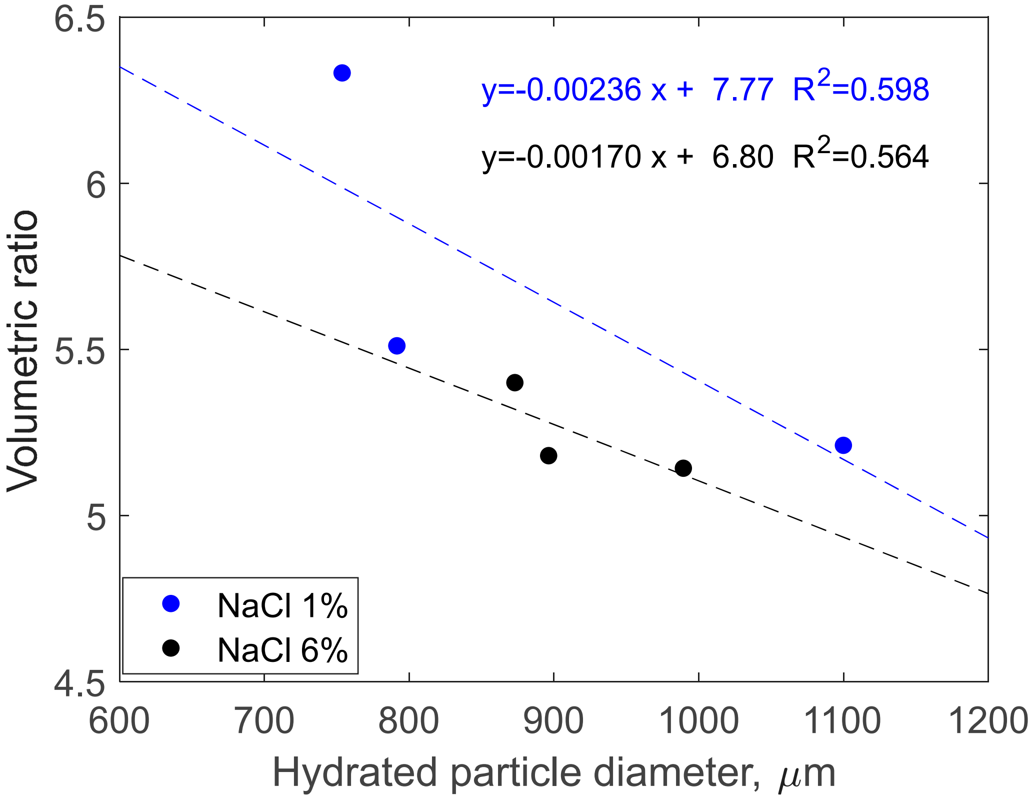

Figure S1. Comparison of DSM volumetric swelling ratio (μm^3/μm^3) with the diameter (μm) of the hydrated starch microsphere in a NaCl 1% salt solution and in a NaCl 6% salt solution.

S1.2 Supplementary data on distances and velocities

(a)

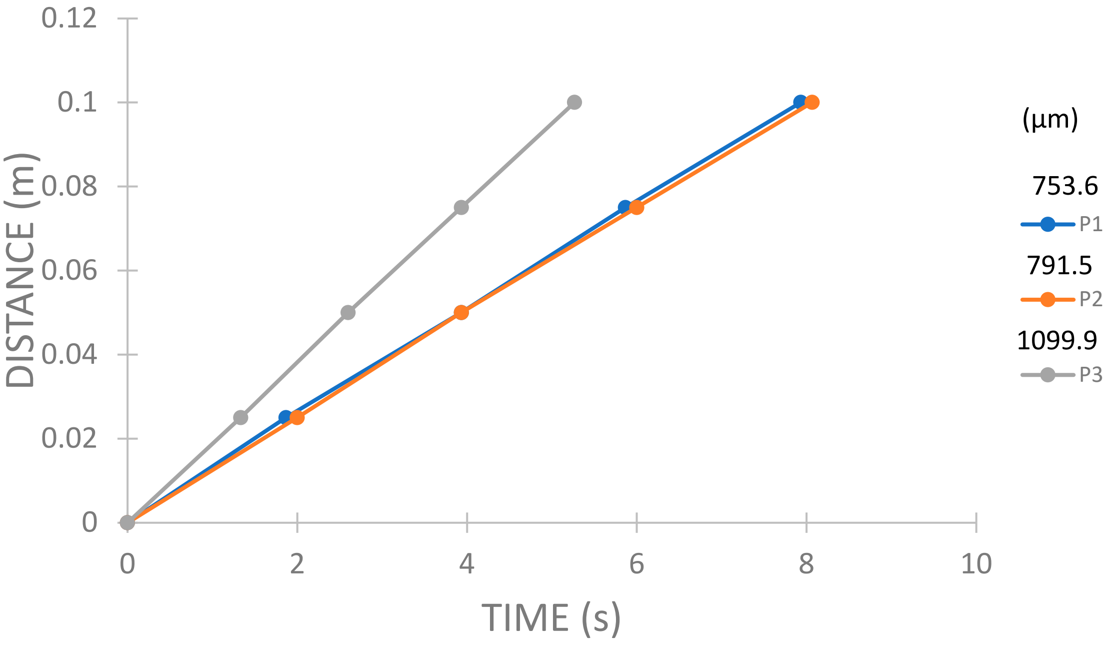

(b)

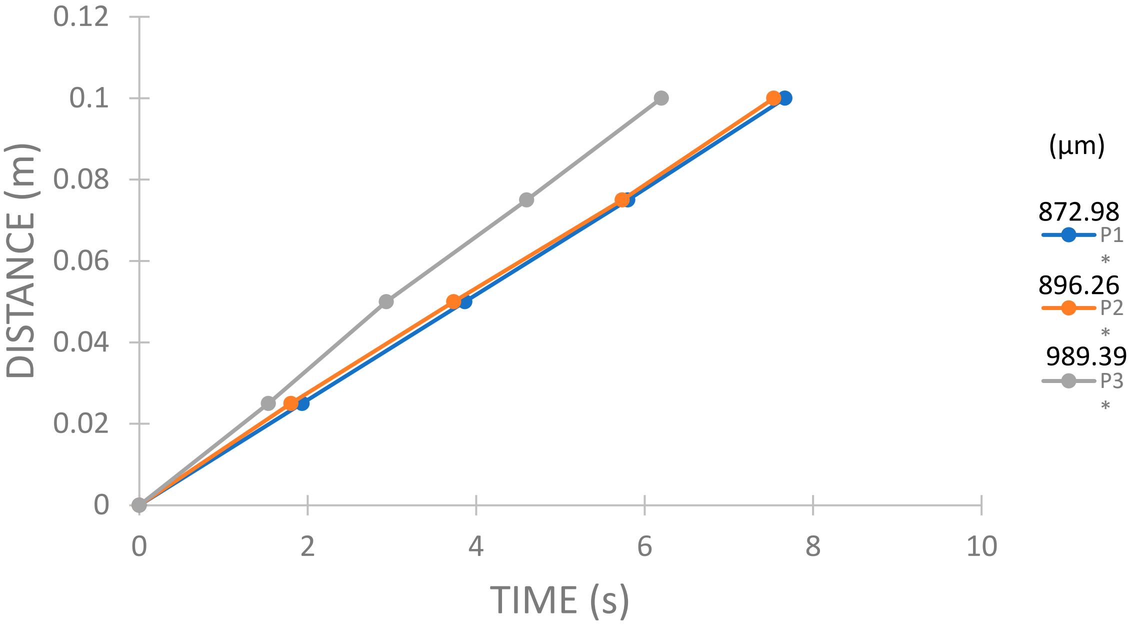

Figure S2. Travelled distances in a NaCl 1% salt solution (a) and in a NaCl 6% salt solution (b).

Table S1. DSM average sedimentation velocity in MQ water.

Particle	Dry particle diameter (μm)	Hydrated particle diameter (μm)	Average velocity (mm/s)
P1	282.3	538.3	6.26
P2	323.0	614.0	8.42
P3	357.9	680.9	9.77
P4	462.7	846.8	13.74
P5	410.3	753.7	12.07
P6	480.1	875.9	13.55
P7	634.4	1126.1	17.80
P8	648.9	1149.4	19.01
P9	683.8	1216.3	19.46

Table S2. Swelling of DSM particles in salt solutions.

		Dry particle diameter (μm)	Hydrated particle diameter (μm)	Volumetric swelling ratio (μm^3/ μm^3)
**NaCl 1 w/V%**	PSS1	407.4	753.7	6.33
	PSS2	448.1	791.5	5.51
	PSS3	634.4	1099.9	5.21
**NaCl 6 w/V%**	PSS1*	497.6	873.0	5.40
	PSS2*	518.0	896.3	5.18
	PSS3*	573.2	989.4	5.14

* In this notation PSS stands for particle in salt solution, the asterisk marks samples in 6% NaCl solution.

Table S3. Sedimentation of DSM particles in salt solutions. Average velocities, Reynolds numbers and drag coefficients.

		Average velocity (mm/s)	Particle density (kg/m^3)	Reynolds number, Re	Drag coefficient, C_D
**NaCl 1 w/V%**	PSS1	12.87	1069.7	10.94	3.89
	PSS2	12.53	1062.4	11.18	3.84
	PSS3	19.01	1063.5	23.59	2.36
**NaCl 6 w/V%**	PSS1*	12.96	1092.7	12.07	3.64
	PSS2*	13.41	1092.7	12.82	3.49
	PSS3*	16.45	1099.1	17.37	2.86

Table S4. DSM average velocity in acid and basic solutions.

Particle		Dry particle diameter (µm)	Hydrated particle diameter (µm)	Average velocity (mm/s)
Acid Solution HCl pH = 4	PAS	503.4	910.8	14.61
Basic Solution NaOH pH = 10	PBS	538.3	969.0	15.04
